# Altered Expression of Specific MicroRNAs in Plasma of Aneurysmal Subarachnoid Hemorrhage Patients

**DOI:** 10.3389/fneur.2022.842888

**Published:** 2022-02-15

**Authors:** Lina Zheng, Xin Zhang, Liping Liu, Yuehua Pu

**Affiliations:** Department of Neurology, Beijing Tiantan Hospital, Capital Medical University, Beijing, China

**Keywords:** subarachnoid hemorrhage, microRNAs, intracranial aneurysm, microarray, genes

## Abstract

**Background:**

Aneurysmal subarachnoid hemorrhage (aSAH) is a life-threatening condition with high disability and mortality. MicroRNAs (miRNAs) are reported to play a modulating role in aSAH. We investigated specific plasma microRNAs (miRNAs) associated with aSAH and gained comprehensive insight into its pathological mechanisms.

**Methods:**

This is a prospective case–control study. We used a two-stage approach, with primary screening and ensuing two-step validation stages. Significantly differentially expressed plasma miRNAs between aSAH patients and neurologically healthy controls were initially screened by microarray analysis. These miRNAs were then validated in two groups of independent cohorts using reverse transcription quantitative real-time polymerase chain reaction assays. Functional annotation of these miRNA targets was performed by Gene Ontology and Kyoto Encyclopedia of Genes and Genomes pathway enrichment analyses.

**Results:**

In the primary screening stage, 14 miRNAs were identified as differentially expressed at a significance level of *P* < 0.05 and fold change >2 between 5 aSAH patients and 5 neurologically healthy controls. In the two validation steps (20 patients vs. 20 control; 40 patients vs. 30 controls), miR-23b-3p, miR-590-5p, miR-20b-5p, miR-142-3p, and miR-29b-3p were found to be significantly down-regulated in patients with aSAH compared with controls. Through these 5 miRNAs, we obtained 32 overlapping target genes, including TGM2, EREG, EDN1, and COL4A1, in three databases that may affect the progression of aSAH. The results of functional annotation revealed mechanisms mainly related to inflammation, smooth muscle cell proliferation and cell adhesion, potentially contributing to the occurrence of aSAH.

**Conclusion:**

We demonstrate that specific miRNAs in plasma, including miR-23b-3p, miR-590-5p, miR-20b-5p, miR-142-3p, and miR-29b-3p, are significantly down-regulated in aSAH patients and may play a modulating role in its progression.

## Introduction

Aneurysmal subarachnoid hemorrhage (aSAH) is a life-threatening condition with high disability and mortality worldwide, accounting for 5–10% of stroke (1). Early identification of aSAH is particularly important. Current diagnostic methods mainly depend on digital subtraction angiography (DSA). However, false negative results may occur in the presence of vasospasm or thrombosis in ruptured aneurysms. In addition, DSA is an invasive operation with underlying risks. Therefore, biomarkers based on the pathophysiology of ruptured aneurysms that distinguish patients with aSAH from a healthy population are urgently needed.

MicroRNAs (miRNAs), molecules 19–25 nucleotides in length, comprise a class of endogenous, non-coding, single-stranded sequences that play critical roles in post-transcriptional regulation by pairing with the 3′ or 5′ -untranslated region (UTR) of target mRNAs and repressing their translation (2). It has been previously demonstrated that miRNAs are stably expressed in the circulating blood at sufficient levels and have great potential for serving as novel, non-invasive biomarkers (3). Recent studies have investigated circulating miRNA profiling as an indicator for cardiovascular diseases (4). Other studies report significant differences in levels of circulating miRNAs at different pathological processes and physiological aneurysm formation stages, including the inflammatory response, endothelial dysfunction and vascular smooth muscle cell phenotypic modulation (5). In this study, we aimed to investigate specific plasma microRNAs (miRNAs) associated with aSAH and to gain comprehensive insight into the pathological mechanisms of this condition.

## Materials and Methods

### Study Design and Subjects

This was a case-control study using a two-stage approach to analyse differential expression of plasma miRNAs between patients with aSAH and age- and sex-matched neurologically healthy controls, including primary screening and ensuing validation stages. We performed microarray analysis in the primary screening stage to identify differentially expressed plasma miRNA profiles. Then, we carried out validation via reverse transcription quantitative real-time polymerase chain reaction (RT-qPCR) assays twice by recruiting two groups of independent cohorts. Based on the two sets of validated significantly expressed miRNAs between patients with aSAH and neurologically healthy controls, we obtained targeted genes and related biological processes through databases.

Patients meeting the following criteria were enrolled prospectively in the current study: 1) age between 20 and 60 years old; 2) aSAH with at least one intracranial aneurysm confirmed by digital subtraction angiography (DSA) in Beijing Tiantan Hospital; and 3) within 10 days after the onset of symptoms. Those with previous cardiovascular diseases, neurological diseases such as dementia and encephalitis, or prior systemic disorders such as severe renal, liver or heart failure and malignancy were excluded. We enrolled neurologically healthy controls from the health physical examination center in Beijing Tiantan Hospital whose age and sex matched the included aSAH patients. First-degree relatives of the controls had no history of intracranial aneurysm or subarachnoid hemorrhage (SAH). Baseline demographic data, including characteristics regarding vascular risk factors such as hypertension, diabetes, smoking, and alcohol consumption, were collected. We collected the time from blood sampling to the aSAH onset in the patients. We also evaluated the imaging features of intracranial aneurysms including aneurysmal number, location, type and size and assessed the Hunt Hess scale (6), modified Fisher scale (7) in the aSAH patients. Moreover, we dichotomized the neurological functional outcomes at discharge of the aSAH patients as good and poor outcomes, which were defined by modified Rankin Scale 0–2 and 3–6, respectively. This study was evaluated and approved by the medical ethics committee of Beijing Tiantan Hospital, Capital Medical University (KY2012-002-01). All participants or their legal representatives provided written informed consent to participate in the study.

### Sample Processing and RNA Isolation

Peripheral venous blood samples were collected into EDTA-containing tubes on the day after admission, processed within 30 min to prevent blood clotting and cell lysis and promptly centrifuged at 2,200 × g for 10 min at room temperature. The plasma was aliquoted into 1.5-mL nuclease-free tubes and stored at −80°C until RNA isolation.

Total RNA, including miRNA, was extracted from venous blood samples using mirVana RNA Isolation Kit (Ambion) according to the manufacturer's standard protocols. The concentration of isolated RNA was measured using a NanoDrop 2000 spectrophotometer (Thermo Scientific, USA), and the RNA integrity was assessed using an Agilent Bioanalyzer 2100 (Agilent Technologies).

### miRNA Microarray Experiment

Qualified total RNA was dephosphorylated, denatured and labeled with Cyanine-3-CTP. The purified labeled RNAs were hybridized onto an Agilent Human miRNA microarray (8*60K, Design ID: 046064) containing 2006 miRNA probes at 55°C for 20 h. After washing, the miRNA microarrays were scanned with an Agilent Scanner G2505C (Agilent Technologies).

### Reverse Transcription Quantitative Real-Time Polymerase Chain Reaction

To validate the microarray-generated plasma miRNAs, we conducted two independent validation stages. In the first stage, we measured levels of miRNAs obtained in the screening stage. In the second stage, we validated the miRNAs identified in the first stage and added miRNA-29b-3p, which was closely related to aneurysm formation (8).

miRNA levels were measured using RT-qPCR, with two reaction processes: reverse transcription (RT) and real-time polymerase chain reaction (qPCR). The RT reaction was performed with GeneAmp® PCR System 9700 (Applied Biosystems, USA); qPCR was performed using a LightCycler® 480 II Real-time PCR Instrument (Roche, Switzerland). Exogenous cel-miR-39 was added as an external control, and expression levels of miRNAs were normalized (9). The reactions were run in triplicate, and we used average values of miRNA levels. The ΔCt (threshold cycle) value was calculated by subtracting the Ct value of the exogenous miRNA (cel-miR-39) from that of the target miRNA. The ΔΔCt value was calculated by subtracting the ΔCt value of the miRNA of the control group from that of the target miRNA. Ultimately, the relative expression levels of miRNAs were calculated via the 2^−ΔΔCt^ method (10). miRNAs were verified in the validation stages when the level differed significantly (*P* < 0.05) and fold change was >2 between the two groups. Investigators performing the whole screening and validation processes above were blinded to the group of samples.

### Functional Annotation

We employed a total of three databases, TargetScan, PITA and microRNAorg, separately to obtain a gene list predicted to be targeted by the differentially expressed miRNAs using GeneSpring13.1 software. Functional annotation of miRNA target genes was performed by Gene Ontology (GO) analysis (11) and Kyoto Encyclopedia of Genes and Genomes (KEGG) pathway enrichment analysis (12). GO was used to uncover the biological functions of the miRNA target genes, including biological process, cellular component and molecular function, and KEGG pathway enrichment analysis was performed to detect potential pathways of the miRNA target genes based on the KEGG pathway database, which is a recognized and comprehensive database including various types of biochemistry pathways. We counted the number of target genes and calculated the statistical significance for each GO and pathway entry. The calculation resulted in a *P*-value, with a smaller value indicating that the target gene was more enriched.

### Statistical Analysis

Baseline characteristics are presented as percentages for categorical variables and means and standard deviations for continuous variables. Chi-square tests and *t*-tests were used to compare categorical and continuous variables between the patients and controls. miRNA levels were compared using the *t*-test. A two-sided *P*-value of <0.05 was considered statistically significant. All statistical analyses were performed using SPSS 24.0 (IBM Corp, Armonk, NY).

## Results

### Clinical Characteristics

**Figure 1:**
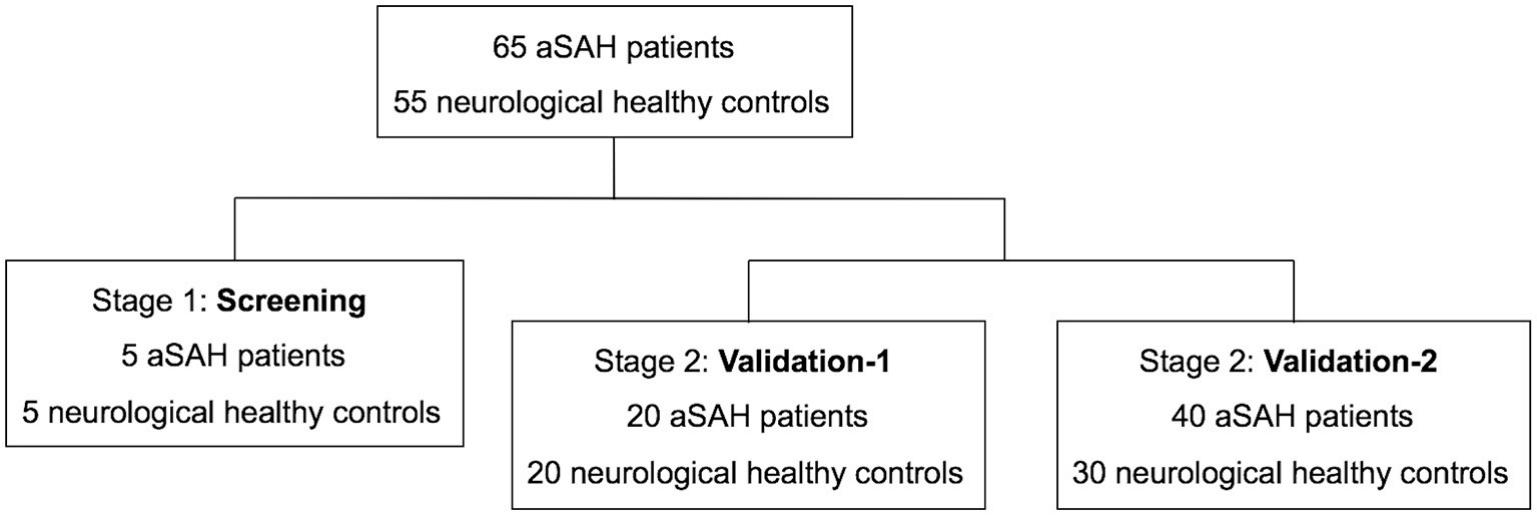
Study flow diagram. aSAH indicates aneurysmal subarachnoid hemorrhage.

A total of 65 aSAH patients and 55 healthy controls were included in the study (Figure 1). The baseline characteristics of the 120 participants in each case–control cohort are shown in Table 1. Among the 65 aSAH patients, 36 (55.4%) were male, and the mean age was 47.28 years. The mean age of the controls was 47.70 years, and 26 (47.3%) of them were male. There were no significant differences between the risk factors between aSAH patients and controls in each case–control cohort. The median time of blood sampling after the aSAH onset was 2 days (interquartile range 2–3). None of the subjects had a history of anticoagulation or antiplatelet drug use.

**Table 1:** General characteristics of patients with aneurysmal subarachnoid hemorrhage and controls^*^.

	**Screening**			**Validation-1**			**Validation-2**		
	**Patients (*n* = 5)**	**Controls (*n* = 5)**	***P*-value**	**Patients (*n* = 20)**	**Controls (*n* = 20)**	***P*-value**	**Patients (*n* = 40)**	**Controls (*n* =30)**	***P*-value**
Age, years	46.80 ± 6.14	46.34 ± 7.13	-	48.50 ± 9.08	47.89 ± 8.83	0.653	46.18 ± 7.64	47.60 ± 7.53	0.637
Sex, male	2 (40.0)	2 (40.0)	-	8 (40.0)	6 (30.0)	0.507	26 (65.0)	18 (60.0)	0.668
**Risk factor**									
Hypertension	2 (40.0)	1 (20.0)	-	10 (50.0)	8 (40.0)	0.525	13 (32.5)	6 (20.0)	0.244
Diabetes mellitus	0 (0.0)	0 (0.0)	-	2 (10.0)	1 (5.0)	1.000	0 (0.0)	0 (0.0)	-
Hyperlipidemia	0 (0.0)	1 (20.0)	-	1 (5.0)	1 (5.0)	1.000	2 (5.0)	2 (6.7)	1.000
Smoking	1 (20.0)	2 (40.0)	-	7 (35.0)	5 (25.0)	0.490	16 (40.0)	11 (36.7)	0.777
Alcoholism	1 (20.0)	1 (20.0)	-	4 (20.0)	3 (15.0)	1.000	13 (32.5)	8 (26.7)	0.598
Time of blood sampling (days after onset)	3 (2–4)	-	-	2 (1-3)	-	-	2 (2-3.5)	-	-

* *Values are mean ± standard deviation or number (%); Student's t-tests were used for comparison of continuous variables and χ^2^-tests for categorical variables*.

There were 74 aneurysms in the 65 aSAH patients. Seven patients had multiple aneurysms: five patients had two aneurysms, and two had three aneurysms. The percentage of anterior communicating arteries was highest, and most aneurysms were saccular and had a size less than 5 mm. Most patients were with grade 2 of the initial Hunt Hess scale (58.5%) and grade 3 of modified Fisher scale (30.8%, Table 2).

**Table 2:** Radiologic and clinical features of patients with aneurysmal subarachnoid hemorrhage^*^.

**Variable**	**aSAH patients (*n* = 65)**
**Aneurysm**	
Number	
1	58 (89.2)
2	5 (7.7)
3	2 (3.1)
**Location**	
ACom	29 (39.2)
PCom	16 (21.6)
ACA	4 (5.4)
ICA	7 (9.5)
MCA	15 (20.3)
PCA	0 (0.0)
VA	1 (1.4)
BA	2 (2.7)
**Type**	
Saccular	54 (73.0)
Irregular	19 (25.7)
Dissecting	1 (1.4)
**Size, diameter (mm)**	
<5	42 (56.8)
5–10	26 (35.1)
11–25	6 (8.1)
>25	0 (0.0)
**Initial Hunt Hess scale**	
Grade 1 (Minimal symptoms)	21 (32.3)
Grade 2 (Severe headache)	38 (58.5)
Grade 3 (Lethargy)	5 (7.7)
Grade 4 (Hemiparesis or stupor)	1 (1.5)
Grade 5 (Coma, posturing)	0 (0.0)
**Modified Fisher scale**	
Grade 0 (No blood)	0 (0.0)
Grade 1 (Thin SAH)	30 (4.6)
Grade 2 (Thin SAH and bilateral IVH)	15 (23.1)
Grade 3 (Thick SAH)	20 (30.8)
Grade 4 (Thick SAH and bilateral IVH)	0 (0.0)
**Outcome, mRS at discharge**	
Good (0-2)	62 (95.4)
Poor (3-6)	3 (4.6)

* *Values are number (%) and median (interquartile range)*.

*aSAH indicates aneurysmal subarachnoid hemorrhage; ACom, anterior communicating artery; PCom, posterior communicating artery; ACA, anterior cerebral artery; ICA, internal carotid artery; MCA, middle cerebral artery; PCA, posterior cerebral artery; VA, vertebral artery; BA, basilar artery and mRS, the modified Rankin Scale*.

### Differently Expressed miRNAs Screened by Microarray Analysis

**Figure 2:**
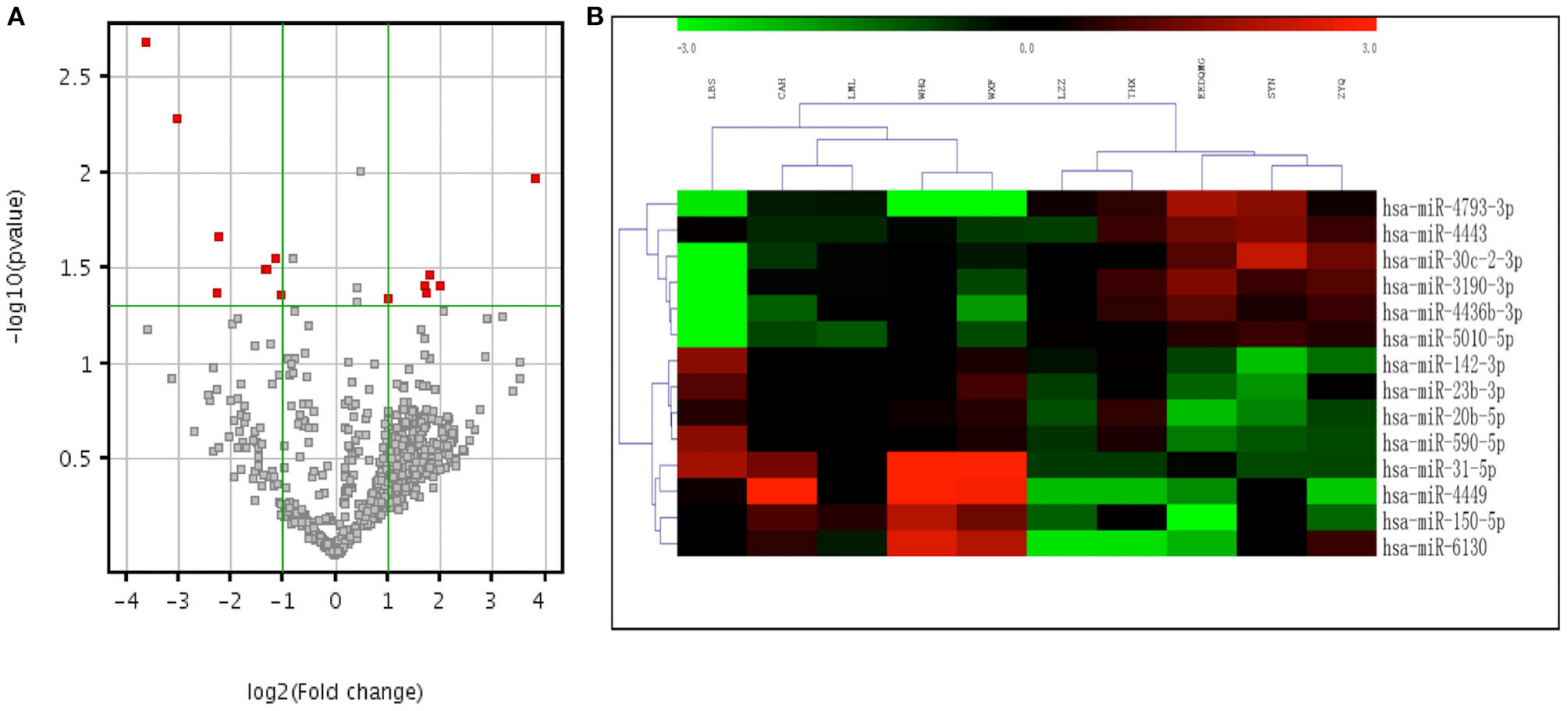
Volcano plot and heatmap expression profiles of differentially expressed miRNAs between aSAH patients and controls in the screening stage. **(A)** Volcano plot of all miRNAs. The –log (P) of each miRNA is plotted against the log2 ratio of aSAH intensity to normal intensity. The green horizontal line indicates the significance level at *P* = 0.05. The green vertical line indicates a fold change level >2. miRNAs that were significantly up-regulated or down-regulated in aSAH patients are indicated by red dots. **(B)** Two-way hierarchical clustering of 14 significant miRNAs. Relatively higher expression levels are represented by red, and relatively lower expression levels are represented by green. miRNAs indicate microRNAs and aSAH, aneurysmal subarachnoid hemorrhage.

The microarray study screened expression levels of approximately 2006 miRNAs isolated from the plasma of 10 participants (5 aSAH patients and 5 controls), who were the first 5 aSAH patients admitted to the hospital and 5 age- and sex- matched neurologically healthy controls. Among 113 miRNAs detected on chips, 14 were differentially expressed between 5 aSAH patients and 5 controls at an adjusted significance level of *P* < 0.05 and fold change >2. Among those, 6 miRNAs were up-regulated and 8 down-regulated in aSAH patients compared to the controls (Table 3). The results of two-way hierarchical clustering of the 14 important miRNAs are presented in a heatmap (Figure 2).

**Table 3:** Plasma miRNAs with significant differences between aSAH patients and controls, as determined by microarray.

**miRNAs**	**Regulation**	**Fold change**	**Sequence**	**Chromosome locus**
miR-4793-3p	Up	14.09	AGCCAGCCAACTCAC	chr3
miR-4436-3p	Up	4.03	TTGTCCACTTCTTCCTG	chr2
miR-5010-5p	Up	3.46	AATTTTGCTCTGCCATCC	chr17
miR-30c-2-3p	Up	3.35	AGAGTAAACAGCCTTCTC	chr6
miR-3190-3p	Up	3.26	TCTCTGGCCGTCTACC	chr19
miR-4443	Up	2.03	AAAACCCACGCCTCC	chr3
miR-4449	Down	−12.36	TGCCTCGCGCAGC	chr4
miR-31-5p	Down	−8.20	AGCTATGCCAGCATCTT	chr9
miR-6130	Down	−4.81	CATACAATCCACTCCCT	chr21
miR-150-5p	Down	−4.72	CACTGGTACAAGGGTTGG	chr19
miR-142-3p	Down	−2.55	TCCATAAAGTAGGAAACACTAC	chr17
miR-20b-5p	Down	−2.44	CTACCTGCACTATGAGCAC	chrX
miR-590-5p	Down	−2.22	CTGCACTTTTATGAATAAGCTC	chr7
miR-23b-3p	Down	−2.08	GGTAATCCCTGGCAATG	chr9

*aSAH indicates aneurysmal subarachnoid hemorrhage*.

### Validation of Candidate miRNAs With RT-qPCR

**Figure 3:**
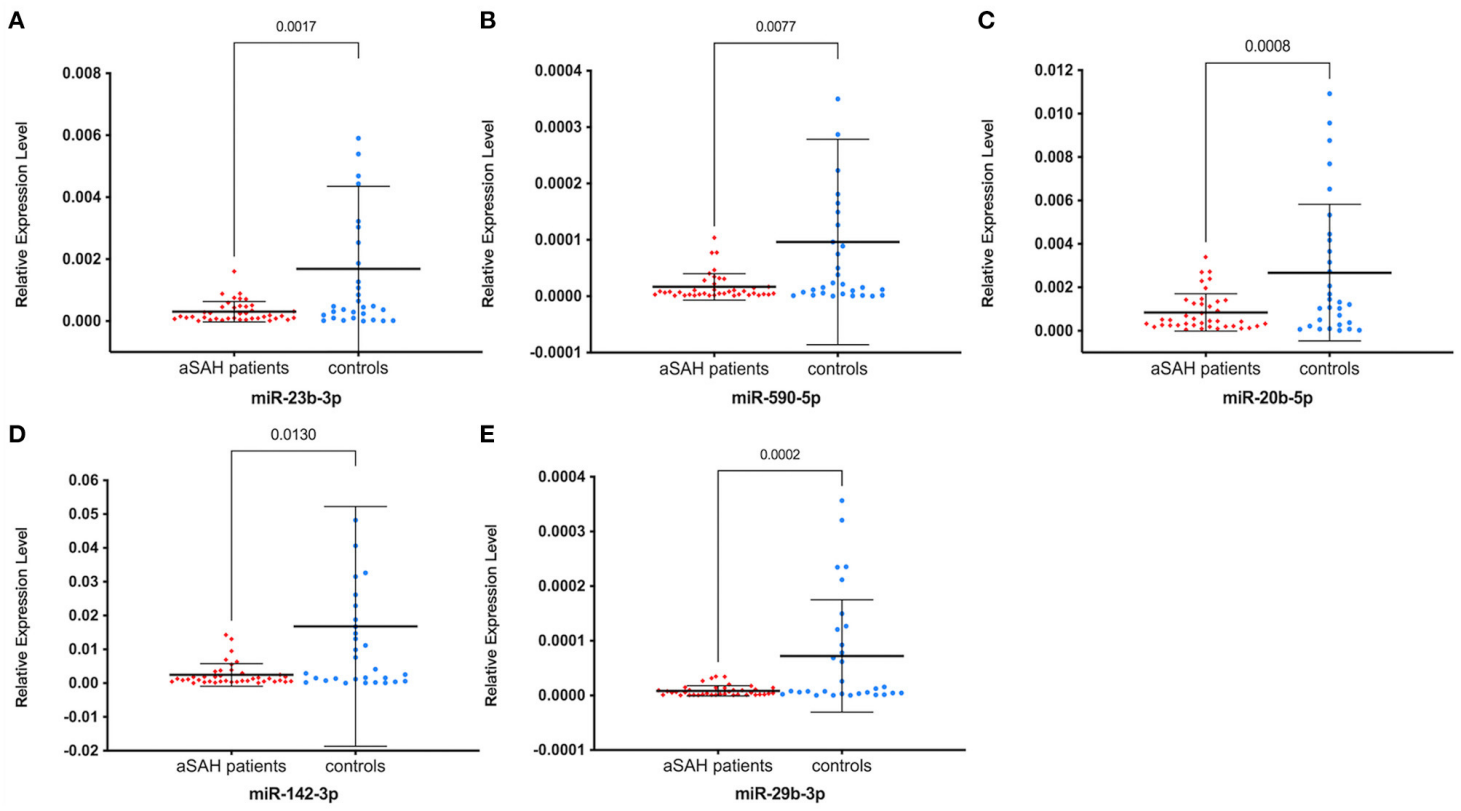
Relative expression levels of five miRNAs between aSAH patients and controls in the validation stage. **(A)** miR-23b-3p; **(B)** miR-590b-5p; **(C)** miR-20b-5p; **(D)** miR-142b-3p; **(E)** miR-29b-3p. Relative expression levels were all significantly lower in aSAH patients than in controls (all *P* < 0.05). miRNAs indicate microRNAs and aSAH, aneurysmal subarachnoid hemorrhage.

We enrolled 40 independent samples (20 aSAH patients vs. 20 controls) in the first small-scale preliminary experiment and validated the 14 initial candidate miRNAs that were significantly expressed in the screening stage by RT-qPCR, and only four miRNAs, hsa-miR-23b-3p, miR-590-5p, miR-20b-5p, and miR-142-3p, were still significantly expressed. According to the results of the experiment, we chose to validate these four miRNAs and added miR-29b-3p, which is reported to be closely related to intracranial aneurysm formation, in another independent cohort of 70 individuals (40 aSAH patients vs. 30 controls) (8). Among the miRNAs identified, all 5 miRNAs were significantly down-regulated in the aSAH patients compared with the controls (all *P* < 0.05; Figure 3), consistent with the preliminary validation experiment results.

### Target Gene Prediction and Functional Annotation

**Figure 4:**
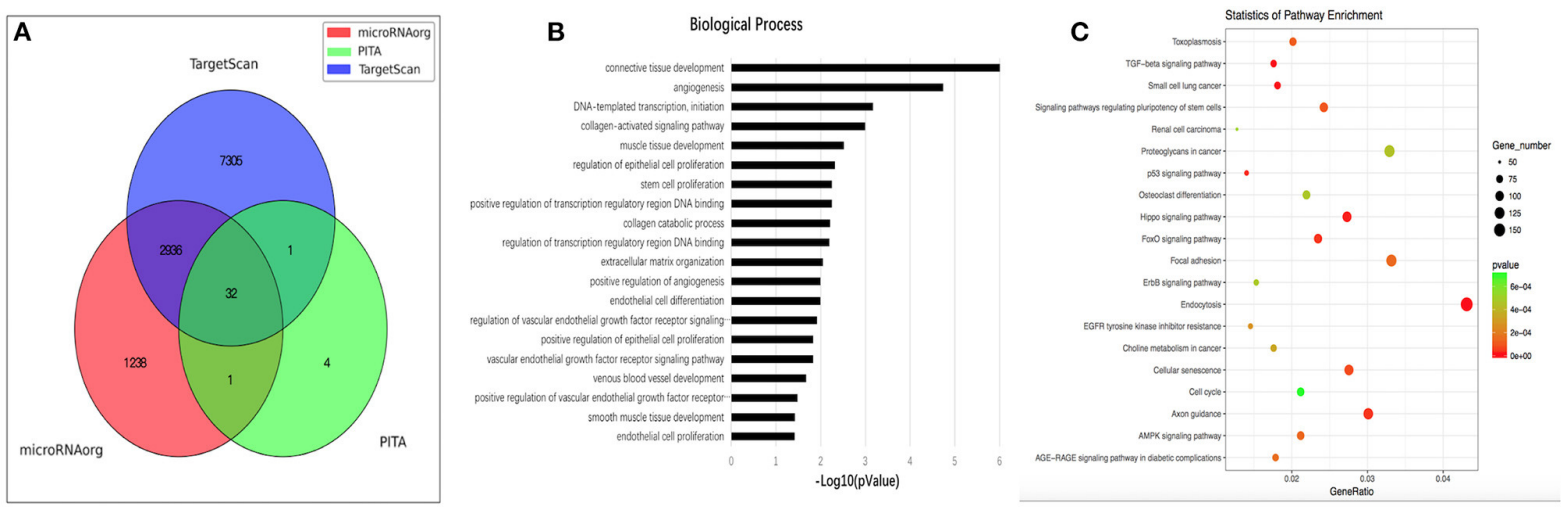
Target genes and results of GO and KEGG analyses. **(A)** 3 targetsbase_venn. Venn diagram of target genes from three databases, TargetScan, PITA, and microRNAorg. **(B)** GO_barplot. Enrichment *P*-value histograms for each GO branch (sorted by *P*-value). **(C)** KEGG_dotplot. A bubble map for the result of KEGG branch enrichment (each KEGG branch is sorted by the *P*-value, taking the first 20 KEGG results). The abscissa is the ratio of the number of genes associated with the KEGG term in the target gene to the number of genes enriched in the KEGG database. The ordinate is the KEGG term. The color scale represents the *P*-value; when the color tends toward red, the *P*-value is smaller. The size of the circle indicates the number of differentially expressed genes in the pathway: the larger the point, the more significant in the gene. GO indicates gene ontology; KEGG, Kyoto Encyclopedia of Genes and Genomes; TGF, transforming growth factor and AMPK, AMP-activated protein kinase.

We identified 32 common target genes based on the intersection of results from three databases, TargetScan, PITA and microRNAorg (Figure 4A), and displayed these 32 genes which were shared by all these three databases in Table 4. Several of these target genes have been reported to be closely related to the formation, growth and rupture of intracranial aneurysms, including transglutaminase 2 (TGM2), epiregulin (EREG), endothelin-1 (EDN1) and alpha 1 type IV collagen (COL4A1).

**Table 4:** Target gene list obtained from three databases.

TGM2	EREG	EDN1	COL4A1	CTNND1	VIM	PDE4B	ME1
KRT10	FNTB	VAMP3	CPEB3	CCNT2	CUL5	ZNF217	LZTFL1
PLEKHA1	GAB1	TMEM59	GTF2A1	CHIC1	PDE8A	EHF	SYPL1
NCK2	MARCKS	LRRC32	C9orf72	CLTA	SKI	MMD	NAV3

We performed GO enrichment analysis to systematically understand the pathological roles of the target genes. The results revealed a broad range of enriched biological processes or molecular function categories (Figure 4B), such as connective tissue development, angiogenesis, signaling pathways, cell proliferation, collagen catabolic process, extracellular matrix organization, and smooth muscle tissue development.

In addition, we carried out pathway analysis using the KEGG database, which indicated the transforming growth factor (TGF)-β signaling pathway (ID: hsa04350) as the most significantly enriched (P = 1.11E-08). Several related pathways involved in sSAH disease were also enriched, including the Hippo signaling pathway (hsa04390), p53 signaling pathway (hsa04115), cellular senescence (hsa04218), AMP-activated protein kinase (AMPK) signaling pathway (hsa04152), focal adhesion (hsa04510), osteoclast differentiation (hsa04380) and cell cycle (hsa04110) (Figure 4C).

## Discussion

The major discovery in our study is that, compared with neurologically healthy individuals, 14 plasma miRNAs were significantly altered in aSAH patients, regardless of the characteristics of the aneurysms, such as location, type and size. In the verification study, 4 miRNAs, hsa-miR-23b-3p, miR-590-5p, miR-20b-5p, and miR-142-3p, expression profiling and supplement one miR-29b-3p, were significantly altered, and we identified 32 putative target genes involved in the progression of aSAH, including TGM2, EREG, EDN1, and COL4A1. Connective tissue development and the TGF-β signaling pathway were the most significantly enriched biological processes and pathways.

Studies on miRNAs associated with intracranial aneurysms are ongoing. Among the five miRNAs screened and validated in our study, it had been previously reported that miRNA-29b and miRNA-23b-3p are differentially expressed in intracranial aneurysm tissues compared with normal tissues (5); in addition, miR-20b-5p might affect the occurrence and development of intracranial aneurysms by regulating actin cytoskeleton biogenesis (13). miR-23b was also found to result in an impaired inhibitory effect on inflammatory cytokine expression and to be closely related to the inflammatory response. It has been demonstrated that downregulation of miR-590-5p promotes angiotensin II-induced endothelial cell apoptosis in aSAH (14). To date, however, there is no report that miR-142-3p is associated with aSAH or intracranial aneurysms, though a mouse study demonstrated that down-regulation of miR-142-3p affects macrophages involved in age-related inflammatory diseases (15). Moreover, some studies have demonstrated that miR-142-3p is a key regulator of the TGF-β-mediated contractile phenotype of vascular smooth muscle cells that acts by inhibiting cell migration (16). Notably, the TGF-β signaling pathway was found to be involved in the progression of aSAH in our study, and we hypothesize that miR-142-3p is involved in the formation of intracranial aneurysms.

We obtained 32 common target genes in total based on intersection of the results from three databases, demonstrating that each miRNA can participate in regulating expression of multiple target genes and that the same gene may also be regulated by several miRNAs. TGM2 is reported to play a key role in positive regulation of the inflammatory response, smooth muscle cell proliferation and cell adhesion and to participate in blood vessel remodeling (17). In addition to TGM2, EREG, coregulated by miR-142-3p, miR-150-5p, miR-20b-5p, and miR-4793-3p, is closely related to the regulation of smooth muscle cell differentiation and proliferation, epidermal growth factor receptor activity and signaling pathways, epithelial cell proliferation and angiogenesis (18, 19). By targeting EDN1, a potent vasoconstrictor produced by endothelial cells, miR-4443 positively regulates smooth muscle cell proliferation and blood vessel morphogenesis (20). Furthermore, COL4A1 appears to be involved in the maintenance of the integrity of the extracellular matrix of the arterial wall, as genetic variation in COL4A1 leads to disruption of collagen type 4 in the basement membrane (21).

By analyzing the results of GO enrichment, we found that a large part of the cause of intracranial aneurysms is related to the structure of the blood vessel wall, including connective tissue development, smooth muscle cell proliferation and endothelial cell inflammation. Endothelial cells are present in the innermost wall of arteries, which are in direct contact with blood. Endothelial cell differentiation and proliferation or inflammation can increase the formation of aneurysms by generating reactive oxygen species (22). Extracellular connective components and vascular smooth muscle cells together form the medial membrane of the vascular wall, which is the main load-bearing structure. Changes in the collagen catabolic process, extracellular matrix tissue and vascular smooth muscle will inevitably lead to a significant decrease in wall tensile strength (23, 24). Although intracranial aneurysms appear to be focal lesions, there is sufficient evidence to suggest that the entire vascular system is altered in patients with intracranial aneurysms. Our study also found that systemic mechanisms such as stem cell differentiation, positive regulation of angiogenesis and venous blood vessel development are involved in the formation of aneurysms.

With regard to target gene-associated pathways, TGF-β signaling was the most significant pathway revealed in KEGG analysis. This finding is consistent with a previous study, which indicated that the TGF-β pathway plays a pivotal role in Loeys-Dietz syndrome by affecting smooth muscle cells, with characteristic arterial tortuosity and aneurysms (25). Another study found that TGF-β signal transduction participates in intracranial aneurysm pathogenesis through endothelial cells, corresponding well with enriched GO categories (26). Furthermore, the Hippo signaling pathway is reported to be essential for angiogenesis and vascular barrier formation and maturation, and cellular senescence is associated with the formation of micro-aneurysm in old-age human retinas (27).

There are some limitations to our study. First, we only enrolled patients with aSAH but none with unruptured intracranial aneurysms. So far, there are relatively few literatures on the effect of aneurysm rupture process on the change of plasma miRNAs. Despite previous studies have shown that the change in miRNA expression levels is consistent in patients with intracranial aneurysms, regardless of their status (either ruptured or unruptured), compared to healthy controls (28), whether the aneurysm rupture process has effect on the expression of plasma miRNAs warrants further prospective and large scale studies to investigate. Second, the sample size of this study was relatively small, especially after splitting into 3 cohorts, which might have led to some bias in the results. In addition, we only analyzed the miRNAs in plasma, which might be influenced by the conditions of peripheral organs after aSAH. Therefore, further studies are needed to explore miRNAs in aSAH.

In conclusion, we demonstrate that miR-23b-3p, miR-590-5p, miR-20b-5p, miR-142-3p, and miR-29b-3p are significantly down-regulated in aSAH patients, which suggests that these 5 miRNAs are associated with aSAH disease. In addition, mechanisms mainly involving inflammation, smooth muscle cell proliferation and cell adhesion potentially contribute to the occurrence of disease. These results provide a foundation for future research to identify genetic differences between the aSAH population and controls, which may lead to a better understanding of the underlying molecular and pathological mechanisms of aSAH and novel therapeutic targets.

## Data Availability Statement

The data cannot be shared as participants were assured that their data would be kept private and confidential to the extent permitted by law, and that only the research team would have access to the data. However, summarized and de-identified data can be available at reasonable request to the corresponding author.

## Ethics Statement

The studies involving human participants were reviewed and approved by the Medical Ethics Committee of Beijing Tiantan Hospital, Capital Medical University. The patients/participants provided their written informed consent to participate in this study.

## Author Contributions

LZ designed the study, analyzed the data, interpreted the findings, and wrote the manuscript. XZ contributed to data collection and analyses. LL provided critical comments and revisions of the manuscript. YP is responsible for the overall content. All authors have read and approved the manuscript.

## Funding

This study was supported by National Natural Science Foundation of China (31271192). The funders of the study had no role in study design, data collection, data analysis, data interpretation, or writing of the report.

## Conflict of Interest

The authors declare that the research was conducted in the absence of any commercial or financial relationships that could be construed as a potential conflict of interest.

## Publisher's Note

All claims expressed in this article are solely those of the authors and do not necessarily represent those of their affiliated organizations, or those of the publisher, the editors and the reviewers. Any product that may be evaluated in this article, or claim that may be made by its manufacturer, is not guaranteed or endorsed by the publisher.
